# Quantitative ^1^H and ^23^Na muscle MRI in Facioscapulohumeral muscular dystrophy patients

**DOI:** 10.1007/s00415-020-10254-2

**Published:** 2020-10-12

**Authors:** Teresa Gerhalter, Benjamin Marty, Lena V. Gast, Katharina Porzelt, Rafael Heiss, Michael Uder, Stefan Schwab, Pierre G. Carlier, Armin M. Nagel, Matthias Türk

**Affiliations:** 1 Institute of Radiology, University Hospital Erlangen, Friedrich-Alexander University Erlangen-Nuremberg (FAU), Maximiliansplatz 3, 91054 Erlangen, Germany; 2 NMR Laboratory, Institute of Myology, Paris, France; 3 NMR Laboratory, CEA/DRF, IBFJ/MIRCen, Paris, France; 4 Department of Neurology, Friedrich-Alexander University Erlangen-Nuremberg (FAU), Erlangen, Germany; 5 Division of Medical Physics in Radiology, German Cancer Research Centre, Heidelberg, Germany; 6 Institute of Medical Physics, Friedrich-Alexander University Erlangen-Nuremberg (FAU), Erlangen, Germany

**Keywords:** Facioscapulohumeral muscular dystrophy, Quantitative MRI, Sodium MRI, Triple quantum filter, Water T_1_

## Abstract

**Objective:**

Our aim was to assess the role of quantitative ^1^H and ^23^Na MRI methods in providing imaging biomarkers of disease activity and severity in patients with Facioscapulohumeral muscular dystrophy (FSHD).

**Methods:**

We imaged the lower leg muscles of 19 FSHD patients and 12 controls with a multimodal MRI protocol to obtain STIR-T_2_w images, fat fraction (FF), water T_2_ (wT_2_), water T_1_ (wT_1_), tissue sodium concentration (TSC), and intracellular-weighted sodium signal (inversion recovery (IR) and triple quantum filter (TQF) sequence). In addition, the FSHD patients underwent muscle strength testing.

**Results:**

Imaging biomarkers related with water mobility (wT_1_ and wT_2_) and ion homeostasis (TSC, IR, TQF) were increased in muscles of FSHD patients. Muscle groups with FF > 10% had higher wT_2_, wT_1_, TSC, IR, and TQF values than muscles with FF < 10%. Muscles with FF < 10% resembled muscles of healthy controls for these MRI disease activity measures. However, wT_1_ was increased in few muscles without fat replacement. Furthermore, few STIR-negative muscles (*n* = 11/76) exhibited increased wT_1_, TSC, IR or TQF. Increased wT_1_ as well as ^23^Na signals were also present in muscles with normal wT_2_. Muscle strength was related to the mean FF and all imaging biomarkers of tibialis anterior except wT_2_ were correlated with dorsal flexion.

**Conclusion:**

The newly evaluated imaging biomarkers related with water mobility (wT_1_) and ion homeostasis (TSC, IR, TQF) showed different patterns compared to the established markers like FF in muscles of FSHD patients. These quantitative biomarkers could thus contain valuable complementary information for the early characterization of disease progression.

**Electronic supplementary material:**

The online version of this article (10.1007/s00415-020-10254-2) contains supplementary material, which is available to authorized users.

## Introduction

Facioscapulohumeral muscular dystrophy (FSHD) is characterized by a typically asymmetric progression of muscle weakness and wasting including muscular inflammation and fibrotic and fatty replacement [1]. Several clinical trials strive to develop treatment and therefore require reliable and accurate tools to monitor muscle response to interventions [2, 3].

Quantitative MRI allows the non-invasive assessment of the structure and function of individual muscles. The majority of MRI studies on muscle dystrophies have measured the fat fraction (FF) within the muscle [4]. However, clinical trials require biomarkers that detect subtle changes in muscle pathology prior to any irreversible fat replacement [5]. Some muscles without visible fatty replacement have shown T_2_ changes reflecting myocyte damage, edema, inflammation and/or hypervascularization [6, 7], and therefore reveal the initial stages of disease expression [8].

The longitudinal relaxation time of water (wT_1_) and ^23^Na MRI have been used to study muscles of patients with other neuromuscular diseases [9–13]. Like transverse relaxation time (T_2_) quantification, water T_1_ mapping enables the characterization of the muscle tissue at the microstructure level. Several studies have already endorsed T_1_ mapping to quantify inflammation, necrosis or fibrosis in the myocardium [14].

In addition, the sodium distribution in the tissue as measured by ^23^Na MRI is a sensitive marker of cell integrity and energy metabolism [15]. ^23^Na MRI revealed an increase of tissue sodium concentration (TSC), which is a volume-weighted average of the intracellular and extracellular sodium concentrations, in the skeletal muscle tissue of Duchenne muscular dystrophy patients and in myotonic dystrophy patients [10–13]. Besides, a partial weighting towards the intracellular sodium can be achieved by relaxation-weighted ^23^Na MRI techniques such as inversion-recovery (IR) imaging [16–18] or triple-quantum filtered (TQF) imaging [19, 20]. Based on differences in T_1_ relaxation times, IR imaging can suppress signal from free sodium ions in fluid such as saline solution [17]. TQF imaging selectively detects signal from sodium ions in environments with restricted motion (e.g., caused by macromolecular interactions) [21]. However, also extracellular sodium ions can contribute to the TQF signal [22].

Quantitative MRI measures such as ^1^H relaxometry and sodium content might therefore be promising early indicators of pathological changes in FSHD. To test this hypothesis, we sought to evaluate the sensitivity of commonly used biomarkers, such as FF, T_2_-weighting, and wT_2_, and novel MRI techniques, such as wT_1_ and ^23^Na MRI, to analyze pathological changes in patients with FSHD.

## Methods

### Study design and subject cohort

The study was approved by the local ethics committee (Number: 10_18 B), and written informed consent was obtained from all participants prior to examination. Between May 2018 and October 2019, we included 19 patients (age 48.1 ± 12.7 years) with genetically confirmed FSHD. 12 age- and sex-matched volunteers (age 46.6 ± 14.2 years) were recruited as controls. Exclusion criteria were contraindications for MRI (e.g., claustrophobia or metallic implants) and an age younger than 18. A neurologist scored manually the strength using the Medical Research Council grading system ranging from 0 (no muscle contraction) to 5 (maximal muscle strength) for the foot dorsal and plantar flexors [23]. MR data were then acquired from the less affected leg or leg without any confounding factor such as former vein thrombosis or lymphatic fistula. In patients with no difference in leg muscle strength, the right leg was scanned. In addition, patients provided a genetic test with the D4Z4 fragment length analyzed by EcoRI-digestion. The neurologist also collected clinically relevant data (self-reported age at disease onset, total disease duration, duration of muscle weakness of lower legs, and medication) by a personal interview and from available medical records.

### MRI acquisitions

MRI was performed on a 3 T whole-body MR system (Magnetom Skyra, Siemens Healthineers, Erlangen, Germany). Total scan time was between 60 and 75 min, including B_0_ shimming and repositioning between the ^1^H and ^23^Na parts of the protocol.

A 15-channel quadrature knee coil (Siemens Healthcare, Erlangen, Germany) was used for ^1^H MRI. The center of the coil was positioned at the biggest circumference of the lower leg. This position was marked to ensure the same positioning for ^23^Na MRI, which was peformed during the second part of the protocol. FF was measured using a 3D 3-point Dixon method (three echo times, 2.75/3.95/5.15 ms; repetition time, 10 ms; flip angle, 3°; 64 slices of 5 mm thickness and with a 1.3 × 1.3 mm^2^ resolution; total acquisition time, 3 min 12 s) [24]. From the same position, short T_1_ inversion recovery T_2_-weighted (STIR-T_2_w) images were acquired with an inversion time of 220 ms to null the fat signal (echo time, 69 ms; repetition time, 6.9 s; flip angle, 145°; 23 slices with 5 mm thickness and 1 × 1 mm^2^ resolution; acquisition time, 3 min 29 s) and to depict possible edematous/ inflammatory/ nectrotic processes within muscle tissue. A multi-spin-echo sequence was acquired with 32 echoes (echo times ranging from 9.5 ms to 304 ms) to determine the wT_2_ (repetition time, 3 s; 5 slices with a 1.4 × 1.4 mm^2^ resolution and 10 mm thickness; acquisition time, 3 min 41 s). We measured wT_1_ with a MR fingerprinting sequence allowing a drastic reduction of acquisition time (train of 1400 spokes; 3 slices with a 1 × 1 mm^2^ resolution and 10 mm thickness; acquisition time, 30 s) [9].

Sodium imaging was performed using a single-resonant (^23^Na) birdcage knee radiofrequency coil (Stark Contrast, Erlangen, Germany) and NaCl reference tubes for ^23^Na signal quantification (20 mM and 40 mM NaCl without and with 5% agarose). Axial ^1^H images were acquired with the body radiofrequency coil using a gradient‐echo sequence to provide anatomical reference for the sodium images and a global flip angle calibration and a manual B_0_ shim using the ^1^H MRI-based B_0_-shimming routine of the manufacturer were performed [13]. The ^23^Na imaging part measured the TSC and intracellular-weighted sodium signal using two relaxation-weighted ^23^Na MRI techniques (IR and TQF imaging). All ^23^Na images were acquired using a density-adapted 3D-radial readout scheme with anisotropic resolution [25]. For TSC assessment, we obtained spin density-weighted ^23^Na images using an echo time of 0.3 ms (repetition time, 120 ms; 5384 projections with 384 radial samples; flip angle, 80°; nominal resolution, 3 × 3 × 15 mm^3^; acquisition time, 10 min 46 s). IR images were acquired with an inversion time of 34 ms to reduce ^23^Na signal originating from free sodium ions (nominal resolution, 4 × 4 × 20 mm^3^; 4760 projections with 384 radial samples; inversion flip angle, 180°; acquisition time, 9 min 50 s) [18]. For 15 FSHD patients and 7 healthy subjects, the ^23^Na protocol was extended with a TQF sequence [26], with the preparation time set to 4 ms as published in [27] (1056 projections with 384 radial samples; zero-filled matrix size, 60 × 60 × 20; nominal spatial resolution, 4 × 4 × 12 mm^3^; acquisition time, 12 min 40 s).

### Data analysis

For the semi-qualitative analysis, a radiologist with 6 years of experience in reading musculoskeletal MRIs evaluated eight different lower leg muscles: tibialis anterior, extensor halluces/digitorum, peroneus, tibialis posterior, gastrocnemius medialis, gastrocnemius lateralis, soleus, and flexor halluces/digitorum longus. The fat replacement of these muscles (fat score) was scored with an established five-point scale ranging from 0 (not-fat replaced) to 4 (completely fat replaced) by Goutallier et al*.* using the first echo of the multi-spin-echo sequence [28]. Similarly, STIR-T_2_w intensity was rated on a five-point scale from 0, normal appearance, to 4, severe involvement of entire muscle, based on the scale described by Wang et al*.* [29]. All imaging slices of each muscle were considered for the grading.

Images for fat fraction and wT_2_ measurement were processed with custom written Python scripts. wT_2_ values were calculated based on a tri-exponential fitting procedure to account for fat replacment [30]. The sodium and wT_1_ data sets were reconstructed offline with custom written MATLAB scripts (MathWorks, Natick, MA, USA). The signal evolution from the MR fingerprinting were matched to a two-component model for water-fat separation and to obtain wT_1_ values [9]. Sodium signals were calibrated using the standard deviation of the background noise and the mean signal of two agarose phantoms (20 and 40 mM NaCl in 5% agarose). ROIs were delineated on the middle slice of the multi-spin-echo images and on the middle slice of the ^1^H anatomical reference image for the ^23^Na part. All ROIs were outlined conservatively within the muscle.

As fat tissue has lower sodium concentrations than muscle tissue [13, 31], we performed a fat correction for the ^23^Na measures to consider partial volume effects of fat replaced muscles as previously described in [13]. Briefly, the estimates of TSC, IR, and TQF in muscle tissue were corrected for the non-negligible sodium signal of fat using the sodium signal of fat and FF of the muscle obtained by the Dixon acquisitions. In the following, the fat-corrected values are presented as TSC, IR, and TQF, respectively. The unit of TSC will be given in mM and refers to the sodium content in mmol per volume of tissue. In addition, the fat-corrected ratios were calculated and will be presented as IR/TSC and TQF/TSC, respectively.

For further analysis, the individual muscles of the FSHD patients were divided into three groups according to their individual intramuscular FF (low: FF < 10%, medium: 10% < FF < 50%, and high: FF > 50%). To analyse the presence of damage, the positive STIR-T_2_w muscles were grouped according to their signal intensity (low: STIR 1, medium: STIR 2, and high: STIR 3 and 4) [29].

### Statistical analysis

Statistical analysis was performed using MATLAB (MathWorks, Natick, MA, USA) and descriptive statistics including boxplots of the average MRI metrics for every ROI were used to evaluate each outcome. Thresholds for aberrant values of any outcome measure were defined as the mean of the control cohort plus two standard deviations. Nonparametric statistics were used as not all the measures from the patient group were normally distributed as assessed by the Lilliefors test. Wilcoxon rank-sum tests compared all MRI parameters for each muscle between dystrophic and control groups. Relationships of MRI parameters with clinical measures of strength were determined using Spearman correlation coefficients for ordinal or non-normally distributed data. False discovery rate (FDR) correction was applied for multiple comparisons [32]. A multiple linear regression model investigated the association between age, duration of leg muscle weakness, and D4Z4 fragment length (independent variables) and the mean FF (dependent variable). The square root transformation was used for variance-stabilization of the mean FF. A *p* value < 0.05 was considered significant for all statistical tests.

## Results

### Clinical characteristics of the study cohort

Nineteen FSHD patients (all FSHD1, mean age, 48.1 ± 12.7 years; age range, 25–67 years; 4 females and 15 males; median disease duration, 19.5 years; range, 2 to > 50 years) and 12 healthy volunteers (mean age, 46.6 ± 14.2 years; age range 25–70 years; 4 females and 8 males) were enrolled for this prospective MRI study. Detailed patient demographic and clinical characteristics are compiled in Table 1. All patients were ambulant at least for short distances (more than 10 m). According to the Medical Research Council score, strength of the ankle dorsal flexors and ankle plantar flexors ranged from 1 to 5 and 3 to 5, respectively. In seven patients, both ankle dorsal flexors and ankle plantar flexors showed normal strength. The mean D4Z4 fragment length was 28 ± 7 (range 17–38) for 18 patients. In patient 15, D4Z4-repeat contraction was not proven in the patient but in his child. However, limited genetic testing confirmed moderate hypomethylation of the distal 4qA-D4Z4-repeat in the patient himself matching the diagnosis of FSHD. Information on their medication are provided in the Supplemental data (Table e-1).

**Table 1:** Patients demographics, clinical data, and qualitative MRI findings of Facioscapulohumeral muscular dystrophy (FSHD) cohort

FSHD patient	Age (years)	Sex	Disease duration (years)	Fragment length by EcoR1-digestion (kb)	Side of examined leg	Duration of lower leg weakness (years)	Dorsal flexion	Plantar flexion	Fat-replaced muscles	Positive STIR-T_2_w muscles
1	66	M	40	38	R	40	4	5	8	0
2	46	M	32	19	L	10	2	3	5	5
3	31	M	9–13	31	L	0	5	5	0	2
4	56	M	16	35 and 32	R	10	2–3	5	4	4
5	65	M	10	27	R	10	2	3	8	9
6	59	F	> 50	24	R	20	1	4	9	1
7	49	M	13	24	L	8	4	5	2	1
8	35	M	21	33	R	0	5	5	0	2
9	58	F	18	36	L	0	5	5	0	0
10	49	M	39	37	L	11	2	5	3	4
11	58	M	5	38	R	5	2	4	4	3
12	67	M	> 50	33	R	40	2	3–4	9	0
13	31	F	21–25	17	R	0	5	5	0	0
14	25	F	15–19	20	L	0	5	5	0	2
15	61	M	2	n/a; *	L	0	5	5	6	5
16	33	M	8	22	R	0	5	5	0	0
17	46	M	30	21	R	30	4	4	3	4
18	45	M	5–30	33	L	2	3	4	0	2
19	55	M	25	20	L	6	2	5	3	5

MRI and clinical data were acquired from the less affected leg (side of examined leg). The duration of muscle weakness for the examined leg is also listed

*R* right leg, *L* left leg, *n/a* not available

^*^Moderate hypomethylation of distal 4qA-D4Z4-repeat

### Qualitative MRI changes

The semi-quantitative scoring revealed that 12 FSHD patients had at least one fat-replaced muscle (Table e-2 in supplemental data). The median fat score of all muscles and patients was 0 [Interquartile range (IQR) 0–3] with a median of 3 affected muscles (IQR 0–6). The gastrocnemius medialis (GM, *n* = 11/19, median fat score 3 [IQR 0–4]) and tibialis anterior (TA, *n* = 10/19, median fat score 2 [IQR 0–4]) were the most affected muscles, whereas the soleus [SOL, *n* = 8/19, median fat score 0 (IQR 0–3)] and gastrocnemius lateralis [GL *n* = 6/19, median fat score 0 (IQR 0–1)] were less often affected by fat replacment and the tibialis posterior (TP) was only mildly fat-replaced in two patients.

STIR-T_2_w imaging showed that 13 out of 18 FSHD patients had at least 1 muscle with hyperintensities. For one patient, no STIR images were acquired due to time constraints. The median STIR score of all muscles and patients was 0 (IQR 0–1) with 2 (IQR 0–4.3) number of STIR-positive muscles. Overall, the GL (STIR 0: *n* = 6/18, STIR 1: *n* = 8/18, STIR 2: *n* = 4/18, STIR 3: *n* = 0/18, STIR 4: *n* = 0/18) was the most frequent STIR-positive muscle, followed by GM (STIR 0: n = 8/18, STIR 1: *n* = 5/18, STIR 2: *n* = 4/18, STIR 3: *n* = 1/18, STIR 4: *n* = 0/18) and TA (STIR 0: *n* = 11/18, STIR 1: *n* = 1/18, STIR 2: *n* = 2/18, STIR 3: *n* = 4/18, STIR 4: *n* = 0/18). Five patients had a STIR-positive SOL (STIR 0: *n* = 13/18, STIR 1: n = 2/18, STIR 2: *n* = 2/18, STIR 3: *n* = 0/18, STIR 4: *n* = 1/18), while no patient had a STIR-positive TP. 12% of these STIR-positive muscles (*n* = 19/162) had no fat replacement (fat score 0). While all muscles of the healthy controls had a fat score of 0, some muscles had a STIR score of 1 (Peroneus *n* = 2/12, GM = 11/12, GL = 11/12, see Table e-3 in supplemental data).

Following the qualitative analysis, we chose to investigate quantitatively muscles that were more affected (GM, TA), moderately affected (SOL), and almost spared (TP) and were big enough for the ROI analysis of ^23^Na MRI.

### Impact of fat on ^23^Na signals

In completely fat-replaced muscles, the sodium signal intensities were close to the signal intensities of subcutaneous fat tissue (Fig. e-1 in supplemental data). Quantification of the sodium signals in the fat-replaced GM muscle of the 59-year-old patient (#6) resulted in 11.2 mM TSC, 0.26 a.u. IR signal, and 0.24 a.u. TQF signal, while the subcutaneous fat yielded 10.6 mM TSC, 0.21 a.u. IR signal, and 0.15 a.u. TQF signal. The sodium values were then corrected for the fat replacement as previously published (TSC_fat_ = 7.0 ± 1.0 mM, IR_fat_ = 0.10 ± 0.04 a.u., and TQF_fat_ = 0.17 ± 0.01 a.u.) [13].

### Quantitative ^1^H and ^23^Na analysis

**Fig. 1:**
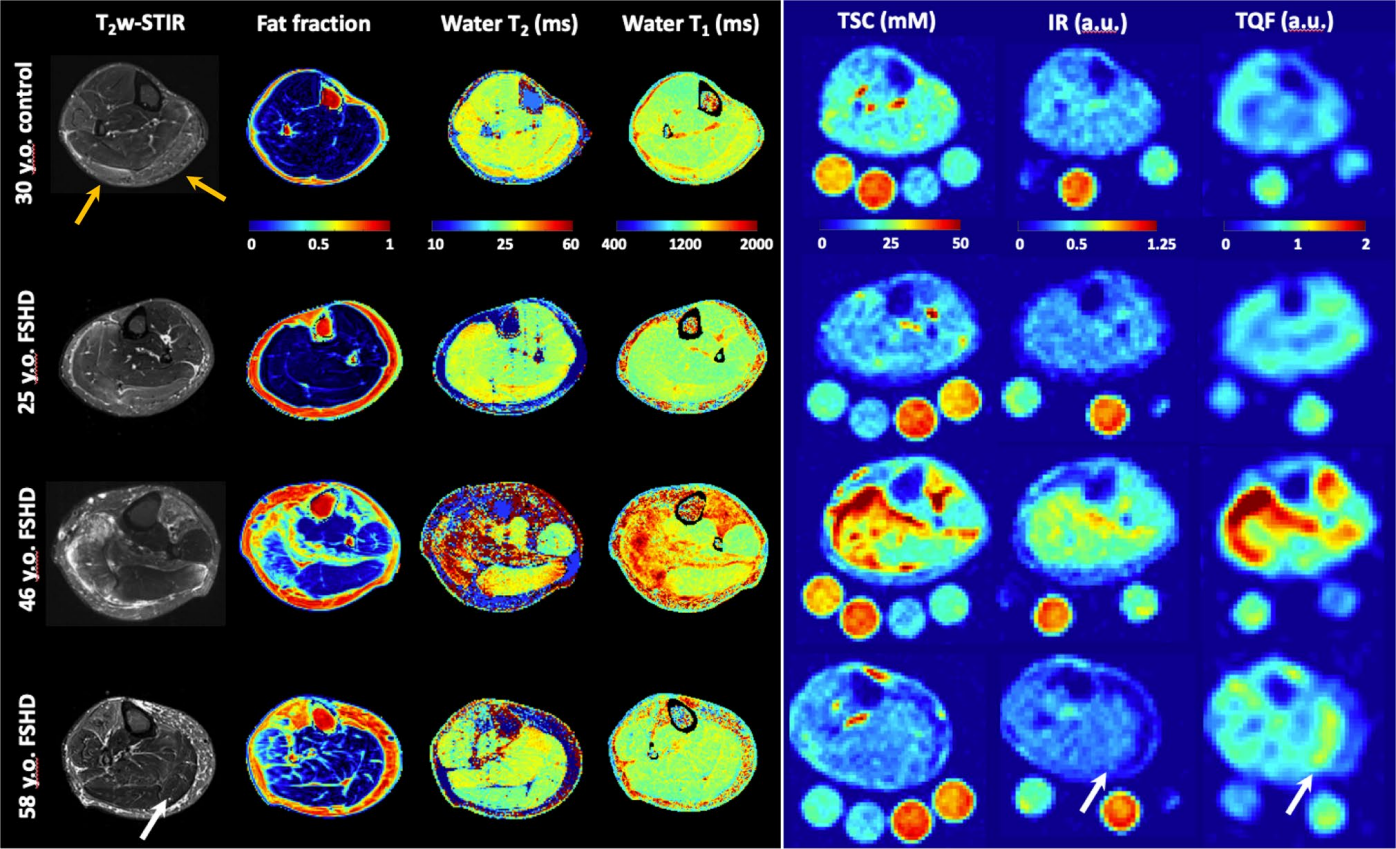
Examples of acquired MR images and maps. T_2_-weighted STIR images are plotted with the corresponding fat fraction, water T_2_, water T_1_, and tissue sodium concentration (TSC), inversion-recovery (IR), and triple-quantum filter (TQF) signal maps in a 30-year-old healthy volunteer (top row) and three Facioscapulohumeral muscular dystrophy (FSHD) patients (2nd–4th row). Note that the 25-year-old patient (#14) presented normal appearing quantitative MRI maps, and only the GM and GL on the STIR image were rated as a one (indicated by orange arrow). On the other hand, fat replacement, moderate to severe edema-like changes, and increased sodium signals were present in selective muscles of the older FSHD patients (disease duration 32 years and 5 years, respectively). While in the 46-year-old patient (#2), the sodium alterations coincided with muscular edema, TQF signal increases without visual edema occurred in the GM of the 58-year-old patient (#11) (see white arrow). Some areas with increased higher TQF signal appeared normal or showed only slightly increased signal intensities in the IR maps. With both methods, the IR and TQF, the ^23^Na signal from vessels and the saline solution phantoms was suppressed sufficiently

Examples of STIR-T_2_w images with the corresponding FF, wT_2_, wT_1_, and sodium maps of one young healthy volunteer in comparison with three FSHD patients of 25, 46, and 58 years of age are shown in Fig. 1. Imaging of the lower leg of the 25-year-old patient (disease onset before school, but no weakness of the legs) showed only two mildly STIR-positive muscles and no increased quantitative MRI measure. In contrast, fat replacement as well as edema-like changes, increased wT_2,_ wT_1_, and sodium signals were present in the 46- and 58-year-old FSHD patients. Both patients exhibit several STIR-positive muscles and increased quantitative indices despite the difference in reported disease duration (32 years and 5 years, respectively) and duration of leg weakness (10 years and 5 years, respectively). Furthermore, the TQF maps showed a different contrast in some muscle compartments compared to IR. Nevertheless, the ^23^Na signal originating from the vessels and saline phantoms was successfully suppressed with both intracellular-weighted sequences.

**Fig. 2:**
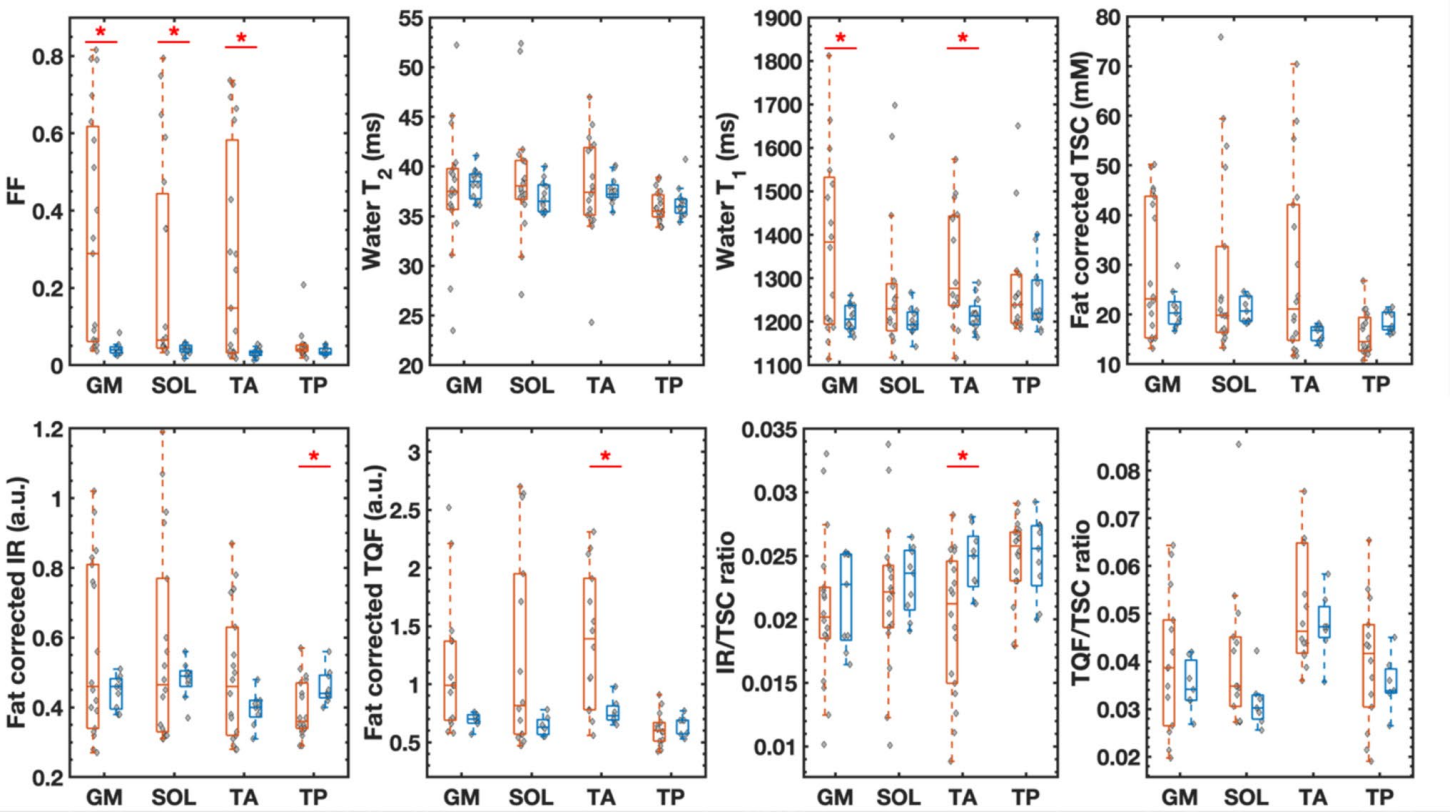
Boxplots showing quantitative ^1^H and ^23^Na MRI measures of analyzed muscles. Fat fraction (FF), water T_1_ and T_2_, and ^23^Na indices of individual muscles in patients with FSHD (orange) and healthy volunteers (blue). The dystrophic muscles were characterized by fatty replacement as well as increased water T_2_, water T_1_ and sodium signals compared to the controls. The decrease in the IR/TSC ratio was significant in the tibialis anterior (TA). In the patient group, the gastrocnemius medialis (GM), soleus (SOL), and TA muscles were more affected than the tibialis posterior (TP) muscle. The middle line in the box depicts the median value and the top and bottom edges of the box the 25th and 75th percentiles of the data, respectively. The whiskers extend to the most extreme data points not considering outliers. *TSC* tissue sodium concentration, *IR* inversion-recovery signal, *TQF* triple-quantum filter signal, *p < 0.05 difference between control and FSHD

For FSHD patients, the fat fraction as assessed in all analyzed 76 muscles by the Dixon method was significantly increased in the GM, SOL, and TA muscles (total *n* = 36/76 muscles) in contrast to the wT_2_ values that remained in the normal range in most of the investigated muscles (Fig. 2). However, several individual muscles of patients showed elevated wT_1_ (*n* = 20/63), wT2 (*n* = 14/74), TSC (*n* = 21/72), IR (*n* = 21/72), and TQF (*n* = 26/60) values compared to controls. Significant increases were observed for the wT_1_ in the GM and TA and the TQF signal in the TA and a significant drop in the IR of the TP and the IR/TSC ratio of the TA. The descriptive statistics of all quantitative MRI parameters are compiled in the Supplemental Material (Table e-4).

**Fig. 3:**
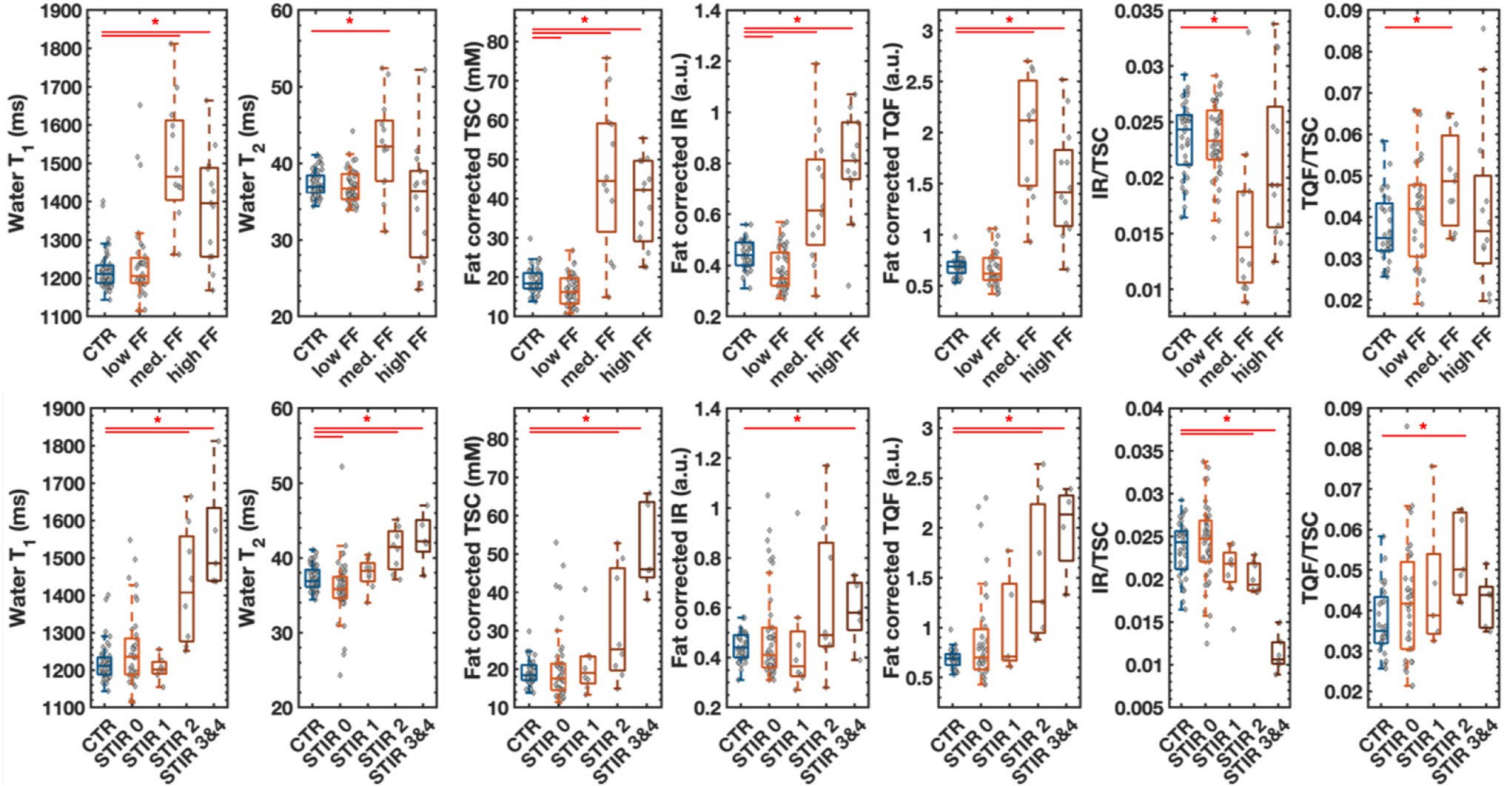
Analysis of muscles grouped according of their FF and STIR score. ^1^H and ^23^Na MRI indices in the leg muscle of FSHD patients (orange-brown) and controls (CTR, blue). The middle line in the box depicts the median value and the top and bottom edges of the box the 25th and 75th percentiles of the data, respectively. The whiskers extend to the most extreme data points not considering outliers. In the top row, the muscles of FSHD patients were gathered in three groups according to their intramuscular fat fraction (low FF: FF < 10%, med. FF: 10% < FF < 50%, and high FF: FF > 50%). Muscles with FF < 10% resembled muscles of healthy controls for all disease activity indices except the TSC and IR. In the bottom row, the muscles of FSHD patients were grouped according to the STIR-T_2_w signal intensity (STIR 0: normal appearance, STIR 1: very mild hyperintensities, STIR 2: mild diffuse/some moderate elevation, and STIR 3 and 4: moderate/severe involvement). Few negative STIR-T_2_w muscles (STIR 0) had increased disease activity indices. Both groups with FF > 10% had higher water T_2_, water T_1_, and sodium values than muscles with FF < 10%. *TSC* tissue sodium concentration, *IR* inversion-recovery signal, *TQF* triple-quantum filter signal, *p < 0.05

To further investigate the relation of fat replacement and STIR scaling with the quantitative indices, we grouped the four muscles (TA, TP, GM, and SOL) in normal appearing, mildly, moderately, and severely affected. The alterations of wT_2_, wT_1_, and the different sodium indices according to the intramuscular fat fraction and STIR scores are plotted in Fig. 3. Muscles with FF < 10% resembled muscles of healthy controls for almost all disease activity indices. Only few muscles with FF < 10% had increased wT_1_ and wT_2_ as compared to controls. The TSC and IR did drop in muscles with low FF (both *p* < 0.05). In contrary, muscles with FF > 10% had significant higher wT_1_, TSC, IR, and TQF values than muscles of the control cohort (all *p* < 0.05). While the IR/TSC ratio dropped for moderately fat-replaced muscles (*p* < 0.05), the TQF/TSC ratio increased for those muscles (*p* < 0.05). STIR-positive muscles showed changes compared to controls for all quantitative disease activity indices, especially in STIR > 1 muscle (STIR 2 and STIR 3 and 4: wT_1_, wT_2_, TSC, TQF, IR/TSC: *p* < 0.05; STIR 3&4: IR: *p* < 0.05; STIR 2: TQF/TSC: *p* < 0.05). Furthermore, some STIR-negative muscles exhibited increases for wT_1_, wT_2_, and all ^23^Na indices compared to controls. Abnormal wT_1_, wT_2_, and ^23^Na indices had an increased STIR score only when FF was low or moderate, since only three muscles had a STIR score > 0 while FF > 50%.

### Correlation of sodium homeostasis and water relaxometry

**Fig. 4:**
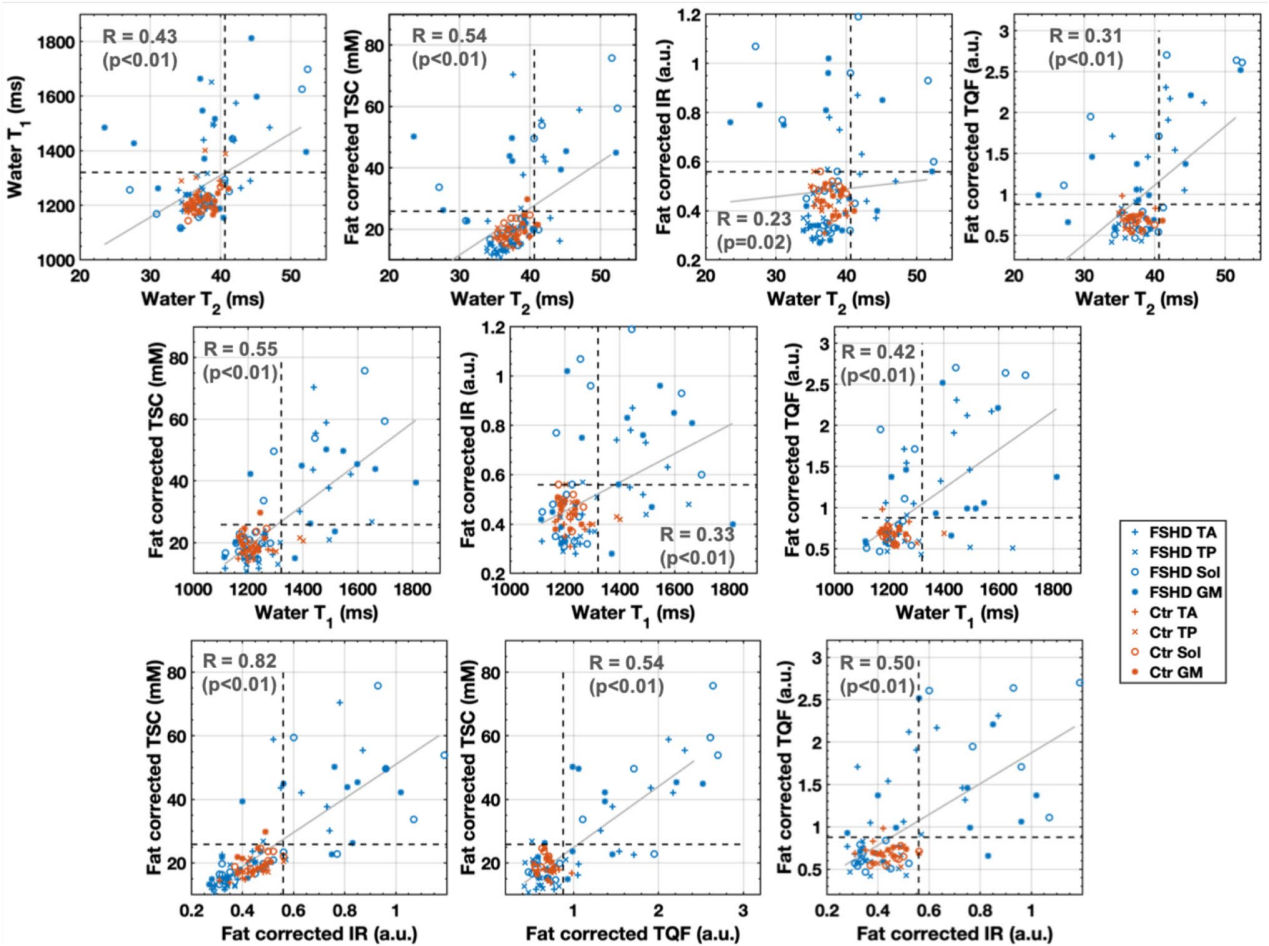
Relationship of water T_1_, water T_2_, and ^23^Na signals. The dashed lines indicate the mean plus two standard deviations of the control values. Aberrant water T_1_, tissue sodium concentration (TSC), inversion-recovery (IR), and triple-quantum filter (TQF) signal could occur also in “quiescent” muscles characterized by normal water T_2_ values. GM was the muscle with most frequent aberrant water T_1_ (*n* = 4), TSC (*n* = 5), IR (*n* = 6), and TQF (*n* = 6) at normal water T_2_, followed by TA (T_1_/TSC/IR/TQF *n* = 3/2/2/3), Sol (T_1_/TSC/IR/TQF *n* = 0/1/3/2), and TP (T_1_/TSC/IR/TQF *n* = 1/1/1/1). TSC correlated better with IR than with TQF, whereas IR did not correlate that well with TQF. *GM* gastrocnemius medialis, *Sol* soleus, *TA* tibialis anterior, *TP* tibialis posterior

The different disease activity indices were correlated to analyze their relationship with each other (Fig. 4). wT_1_ showed stronger correlation with TSC (*R* = 0.55, *p* < 0.01) than with wT_2_ (*R* = 0.43, *p* < 0.01). wT_2_ correlated the most with TSC (*R* = 0.54, *p* < 0.01), before TQF (*R* = 0.31, *p* < 0.01) and IR (*R* = 0.23, *p* = 0.02). Also, elevated wT_1_, TSC, IR, and TQF signals could occur at normal wT_2_. These alterations at normal wT_2_ occurred in all four muscles, while the GM was the most prominent muscle to have increased wT_1_ or ^23^Na indices. These observations were not identical with a positive STIR-T_2_w muscle; eight muscles had at least one increased index at normal wT_2_ and were STIR-negative, whereas seven other muscles were STIR-positive with at least one increased value at normal wT_2_. Also, none of these muscles belonged to a clinical positive patient with a weakness of the lower leg. Furthermore, the TSC signal showed the strongest correlation with the IR (*R* = 0.82, *p* < 0.01) followed by the correlation with the TQF signal (*R* = 0.54, *p* < 0.01), whereas the TQF correlated less with the IR (*R* = 0.50, *p* < 0.01). Multiple comparison correction did not change the significance level of the correlations. Correlations and corrected *p* values are compiled in the Supplemental Material (Table e-5).

### Association of MRI data and clinical measures

In addition, we aimed to describe the concordance between MRI measures and assessment of muscle function and clinical data. Our multiple linear regression model, which included duration of leg muscle weakness, age, and fragment length as independent variables (*n* = 18 FSHD patients), showed that age and duration of leg weakness had a significant influence (coefficient 0.010 with *p* < 0.001 and 0.008 with *p* = 0.002, respectively) on the square root transformed average FF. An influence of the fragment length on the fat fraction was also suggested (coefficient − 0.006 with *p* = 0.088). Our model explained 84% of the variability of the FF (adjusted *R*^2^ = 0.835). The muscle strength was related to the mean FF of the GM and SOL for the plantar flexion (*R* =  − 0.52, *p* = 0.02) and the FF of TA for the dorsal flexion (*R* =  − 0.79, *p* < 0.01) (Fig. e-2 in supplemental data). Furthermore, all disease indices of TA except wT_2_ were correlated with dorsal flexor strength (wT_1_ *R* = −0.67, *p* < 0.01; wT_2_ *R* =  − 0.25, *p* = 0.32; TSC *R* =  − 0.75, *p* < 0.01; IR *R* =  − 0.69, *p* = 0.02; TQF R =  − 0.59, *p* = 0.03).

Finally, seven patients showed no decline in strength (dorsal flexion 5 and plantar flexion 5). However, imaging of one of these patients (61 years, #15) revealed that the GM and SOL are completely or partly replaced by fat. Moreover, the part of the SOL that was spared from fat replacement was severely affected by edema with alterations in all quantitative disease activity indices (Fig. e-3 in supplemental data). One patient (25 years, #14) without strength decline exhibited increased TQF/TSC ratio in the TP and TA. The rest of this subgroup did not show any abnormal quantitative measures. Conversely, another patient (45 years, #18) had decreased muscle strength (dorsal flexion 3 and plantar flexion 4) while the muscles were not replaced by fat and only the TA showed an increased TQF.

## Discussion

In this study, we systematically characterized the status of calf muscle tissue in FSHD patients using different quantitative MRI techniques. To do so, we combined different ^1^H and ^23^Na MRI methods to assess fat replacement, STIR-positive, and normal appearing muscles in patients with FSHD. For the first time, ^23^Na MRI as well as wT_1_ measurements were reported for FSHD revealing the presence of sodium and wT_1_ anomalies in skeletal muscle tissue of FSHD patients.

Involvement of lower leg muscles in patients with FSHD, especially of the GM and TA, is a typical clinical and MRI finding and has also been observed in our study [33–36]. We showed as previously reported that fatty replacement of the muscle increases with age and with disease duration and correlates inversely with muscle flexor strength [37]. While the TA is the sole muscle involved in the dorsal flexion, the plantar flexor comprises the GM, GL, and SOL. The involvement of three muscles could allow a compensation of an affected muscle by the others and thus explains the observed weaker relation with the measured plantar flexor strength than with the dorsal flexor in our study. Furthermore, for two patients, muscle strength was not in accordance with fat replacement.

The muscles of FSHD patients were mostly either high- or low-fat replaced; few were moderately fat replaced (10% < FF < 50%). In a longitudinal study, FF quantification detected muscle changes in FSHD patients without any loss of muscle function [7]. Since fat measurement (qualitative or quantitative) are capturing the muscle wasting too late, scientists are seeking for other earlier markers before clinical involvement of the leg muscles. In concordance with other studies, STIR-T_2_w lesions were present in some muscles before significant fat replacement [6, 38]. Previously, the presence of these hyperintense lesions was linked to increased fat deposition at follow-up [38]. In line, our FSHD cohort showed several muscles with STIR-T_2_w hyperintensities, however also almost all controls had at least one STIR-positive calf muscle providing false-positive findings and thus suggesting a hypersensitivity of STIR-T_2_w imaging. Thus, we wanted to provide quantitative markers of active disease injury by assessing the relaxation times of the muscles. Isolated muscles in our cohort showed increased wT_1_ and wT_2_ values without significant fat replacement. Furthermore, these quantitative variables were also increased in STIR-negative muscles. The sensitivity of the relaxation times to the underlying disease activity seems to differ from each other, as some muscles had increased wT_1_ values at normal wT_2_ values.

^23^Na MRI was able to detect sodium anomalies in skeletal muscle tissue of FSHD patients, but the cause of changes in sodium signals is not fully clear. Water relaxometry was also able to detect changes related to the water mobility in the present FSHD cohort. For the TSC as for wT_1_ and wT_2_, the extracellular and intracellular compartments contribute both to the signal, and derangements such as edema, fibrosis, inflammation, or membrane leakiness could contribute to altered signal. Edema as observed on the STIR-T_2_w images concurrently occurred with higher TSC, wT_1_, and wT_2_ values. Although all three indices are non-specific and reflect inflammation, edema, necrosis, or fibrotic changes, they could show different behavior in the same muscle. While wT_2_ signal as extracted from the T_2_ signal decay represents essentially the intracellular wT_2_, TSC and wT_1_ likely capture more the vascular compartment. Moreover, corticosteroid treatment has been shown to decrease the wT_2_ values at least in Duchenne muscular dystrophy patients [39]. Similar effects from the patients’ medication could have influenced water relaxation times and ^23^Na homeostasis in our study; nevertheless, medical histories did not reveal any medication obviously affecting the sodium or wT_1_ imaging.

To better understand the origin of sodium signal changes, we measured the intracellular-weighted sodium signal by two different MRI methods. First, the IR sequence, which is the more established method, reduced the ^23^Na signal from extracellular edema and blood vessels exploiting the differences in T_1_ of sodium [18, 40]. Secondly, TQF imaging suffers from a poorer signal-to-noise ratio and detects only signal from sodium ions experiencing slow isotropic or anisotropic motion [41]. Nevertheless, both methods can only achieve a weighting towards the intracellular compartment [13, 42–44]. Irrespective of the technique, our FSHD patients showed high intracellular-weighted signals in some muscle compartments, thus pointing to a role of increased intracellular sodium in FSHD especially amid muscle wasting (10% < FF < 50%). Since IR and TQF are more weighted to the intracellular compartment while TSC is more related to the vascular compartment, we expected the IR and TQF to be closely correlated with each other and less correlated with TSC. However, the correlation between IR and TQF was the weakest of all and IR was very strongly correlated to TSC. This observation could be related to the mechanism of the different techniques. While IR is optimized to suppress signal originating from sodium ions in free solution such as saline solution, the TQF sequence filters the signal of nuclei (ions) involved in slow molecular motion. The latter pulse sequence probably shows a stronger dependency on the strength of the motion restriction (i.e., change of correlation time) [45]. Changes in the environment such as myocyte damage or fibrosis could change the interactions of sodium with the environment. However, the signal behavior of TQF imaging is still subject of current research.

Muscles of FSHD patients without fat replacement (FF < 10%) had normal sodium levels, in contrast what has been reported for Duchenne patients [13]. In addition, wT_1_ and wT_2_ values were in the normal range for most FF < 10% muscles. This might be explained by the muscle pathology and the disease course of FSHD. A normal-appearing muscle of a Duchenne muscular dystrophy patient is undergoing waves of necrosis and regeneration while a non-fatty replaced FSHD muscle might be even close to normal. In Duchenne muscular dystrophy, the affected dystrophin complex interacts also with the sodium channel, which could also contribute to the difference in sodium imaging between normal-appearing muscles of a Duchenne patient and FSHD muscles. Since most participating FSHD patients were already in a more advanced stage of the disease, the affected muscles were already replaced at least partly by fatty tissue. A 4-month follow-up MRI study has suggested that fat replacement occurs first distally in the muscle with altered metabolic profile, but then rapidly progresses over the whole muscle [37]. This might create a challenge to detect the starting point of the dystrophic changes.

This report presents the initial results of our FSHD study; we showed that sodium anomalies and ^1^H relaxation time differences are present in FSHD patients and can be measured in vivo on a clinical 3 T scanner. Though other muscles are also involved, we only examined the lower leg due to the geometry of our ^23^Na coil. Time constraints prevented us from acquiring TQF and STIR-T_2_w images from all subjects. Thus, the number of examined muscles differ between MRI measures to due missing data points (TQF and wT_1_ not acquired for all patients, sodium acquisition failed once due to problems with coil) or because the fitting procedure failed due to high fat infiltration (wT_2_). Furthermore, results regarding disease duration should be interpreted with caution since the retrospective determination of the onset presented a challenge despite medical records and self-reports.

## Conclusion

These results demonstrated that wT_1_ and sodium alterations are present in the muscles of FSHD patients compared with healthy controls. Specifically, fat replaced muscles showed changes in wT_1_, TSC, and intracellular-weighted ^23^Na signals. Further work is needed to investigate the time scale of these disturbances in FSHD patients and the potential of quantitative ^1^H and ^23^Na MRI as sensitive biomarkers for intervention. Nevertheless, our study shows that quantitative MRI could be more accurate than visual scores for the status of FSHD, which might be a promising tool for further interventional studies.

## Electronic supplementary material

Below is the link to the electronic supplementary material.

Supplementary file1 (DOCX 937 kb)

## Abbreviations

FF: Fat fraction
FSHD: Facioscapulohumeral muscular dystrophy
GL: Gastrocnemius lateralis muscle
GM: Gastrocnemius medialis muscle
IQR: Interquartile range
IR: Inversion recovery
SOL: Soleus muscle
STIR-T_2_w: Short T_1_ inversion recovery T_2_-weighted
wT_1_: Longitudinal relaxation time of water
wT_2_: Transverse relaxation time of water
TA: Tibialis anterior muscle
TP: Tibialis posterior muscle
TQF: Triple quantum filter
TSC: Tissue sodium concentration
