# Supplementary material for: Quantitative 1H and 23Na muscle MRI in Facioscapulohumeral muscular dystrophy patients
Source: J Neurol. 2020 Oct 12;268(3):1076–87. doi: 10.1007/s00415-020-10254-2 (PMC7914168; doi:10.1007/s00415-020-10254-2)
Supplement: Supplementary file 1 — Supplementary file1 (DOCX 937 kb) [file 415_2020_10254_MOESM1_ESM.docx]

**Journal of Neurology**

**Quantitative ^1^H and ^23^Na muscle MRI in**

**Facioscapulohumeral muscular dystrophy patients**

*Teresa Gerhalter, PhD ^1^ ([0000-0001-9734-4632](https://deref-gmx.net/mail/client/Iq0Lnn72sOE/dereferrer/?redirectUrl=http%3A%2F%2Forcid.org%2F0000-0001-9734-4632)), Benjamin Marty, PhD ^2,3^ ([0000-0002-4983-647X](https://orcid.org/0000-0002-4983-647X)), Lena V. Gast, MSc ^1,6^( [0000-0002-4599-1122](https://orcid.org/0000-0002-4599-1122)), Katharina Porzelt, MD ^4^, Rafael Heiss, MD ^1^ ([0000-0002-2897-5411](https://orcid.org/0000-0002-2897-5411)), Michael Uder, MD ^1^, Stefan Schwab, MD ^4^, Pierre G. Carlier, MD ^2,3^, Armin M. Nagel, PhD ^1,5,6^ ([0000-0003-0948-1421](https://orcid.org/0000-0003-0948-1421)), and Matthias Türk, MD ^4^ ([0000-0001-9812-3794](https://orcid.org/0000-0001-9812-3794))

*^1^Institute of Radiology, University Hospital Erlangen, Friedrich-Alexander University Erlangen-Nuremberg (FAU), Erlangen, Germany*

*^2^NMR laboratory, Institute of Myology, Paris, France*

*^3^NMR laboratory, CEA/DRF/IBFJ/MIRCen, Paris, France*

*^4^Department of Neurology, Friedrich-Alexander University Erlangen-Nuremberg (FAU), Erlangen, Germany*

*^5^Division of Medical Physics in Radiology, German Cancer Research Centre, Heidelberg, Germany*

*^6^Institute of Medical Physics, Friedrich-Alexander University Erlangen-Nuremberg (FAU), Erlangen, Germany*

*Corresponding author / institution from which the work originated

E-mail: [teresagerhalter@gmx.at](mailto:teresagerhalter@gmx.at)

**Supplemental Data**

**Table e-1: Patients demographic and medication information.**

| **FSHD patient** | **Age (years)** | **Sex** | **Disease duration (years)** | **Medication** |
| --- | --- | --- | --- | --- |
| **1** | 66 | m | 40 | Acetylsalicylic acid; Metformin; Liraglutid; Ivabradine; Metoprolol; Insulin; Spironolactone; Ramipril; Bimatoprost/Timolol; Brinzolamide |
| **2** | 46 | m | 32 |  |
| **3** | 31 | m | 9-13 |  |
| **4** | 56 | m | 16 | Allopurinol; Acetylsalicylic acid; Magnesium; Olmesartan; Amlodipine; Hydrochlorothiazide |
| **5** | 65 | m | 10 | Ramipril; Glimepiride |
| **6** | 59 | f | >50 | Venlafaxine |
| **7** | 49 | m | 13 |  |
| **8** | 35 | m | 21 |  |
| **9** | 58 | f | 18 | Rivaroxaban |
| **10** | 49 | m | 39 |  |
| **11** | 58 | m | 5 | Fentanyl; Oxycodone; Acetylsalicylic acid; Atorvastatin; Bisoprolol; Ramipril; Amlodipine |
| **12** | 67 | m | >50 | Metformin; Acetylsalicylic acid; Bisoprolol |
| **13** | 31 | f | 21-25 | oral contraceptive pill |
| **14** | 25 | f | 15-19 | oral contraceptive pill |
| **15** | 61 | m | 2 | Moxonidine; Acetylsalicylic acid; Dihydralazine; Allopurinol; L-thyroxine; Candesartan; Metformin;Bezafibrate; Amlodipine; Torasemide; Metoprolol; Atorvastatin; Insulin |
| **16** | 33 | m | 8 |  |
| **17** | 46 | m | 30 | Candesartan; Hydrochlorothiazide; Allopurinol |
| **18** | 45 | m | 5-30 | Levodopa; Etoricoxib; Cannabidiol |
| **19** | 55 | m | 25 |  |

**Table e-2**: **Semiquantitative scores for lower leg muscles of FSHD patients.** The fat replacement was scored with the five-point scale by Goutallier *et al.* and STIR-T_2_w intensities were rated on a scale from 0, normal appearance, to 4, severe involvement of entire muscle. No STIR-T_2_w images were acquired for patient #5. TA… tibialis anterior, EHL/EDL… extensor halluces/digitorum, PER… peroneus, TP… tibialis posterior, GM… gastrocnemius medialis, GL… gastrocnemius lateralis, SOL… soleus, FHL… flexor halluces longus, FDL… flexor digitorum longus

| **FSHD patient** | **TA**  Fat/STIR | **EHL/DL**  Fat/STIR | **Per**  Fat/STIR | **TP**  Fat/STIR | **GM**  Fat/STIR | **GL**  Fat/STIR | **SOL**  Fat/STIR | **FHL**  Fat/STIR | **FDL**  Fat/STIR |
| --- | --- | --- | --- | --- | --- | --- | --- | --- | --- |
| **1** | 4/0 | 1/0 | 1/0 | 0/0 | 4/0 | 4/0 | 4/0 | 4/0 | 4/0 |
| **2** | 4/3 | 4/3 | 0/0 | 0/0 | 3/2 | 1/2 | 4/2 | 0/0 | 0/0 |
| **3** | 0/0 | 0/0 | 0/0 | 0/0 | 0/1 | 0/1 | 0/0 | 0/0 | 0/0 |
| **4** | 3/3 | 3/3 | 1/0 | 0/0 | 4/2 | 0/2 | 0/1 | 0/0 | 0/0 |
| **5** | 4/- | 4/- | 1/- | 0/- | 4/- | 1/- | 2/- | 1/- | 1/- |
| **6** | 4/0 | 4/0 | 1/0 | 2/0 | 4/0 | 3/2 | 3/0 | -/0 | -/0 |
| **7** | 0/0 | 0/0 | 1/0 | 0/0 | 4/1 | 0/1 | 0/0 | 0/0 | 0/0 |
| **8** | 0/0 | 0/0 | 0/0 | 0/0 | 0/1 | 0/1 | 0/0 | 0/0 | 0/0 |
| **9** | 0/0 | 0/0 | 0/0 | 0/0 | 0/0 | 0/0 | 0/0 | 0/0 | 0/0 |
| **10** | 3/3 | 1/3 | 0/0 | 0/0 | 3/3 | 0/1 | 0/1 | 0/0 | 0/0 |
| **11** | 4/1 | 4/1 | 1/0 | 0/0 | 0/2 | 0/0 | 1/0 | 0/0 | 0/0 |
| **12** | 4/0 | 4/0 | 4/0 | 1/0 | 4/0 | 4/0 | 4/0 | 4/0 | 4/0 |
| **13** | 0/0 | 0/0 | 0/0 | 0/0 | 0/0 | 0/0 | 0/0 | 0/0 | 0/0 |
| **14** | 0/0 | 0/0 | 0/0 | 0/0 | 0/1 | 0/1 | 0/0 | 0/0 | 0/0 |
| **15** | 2/2 | 0/0 | 0/0 | 0/0 | 4/0 | 1/2 | 3/4 | 1/0 | 1/0 |
| **16** | 0/0 | 0/0 | 0/0 | 0/0 | 0/0 | 0/0 | 0/0 | 0/0 | 0/0 |
| **17** | 0/2 | 0/2 | 0/1 | 0/0 | 4/0 | -/1 | 4/0 | 0/0 | 0/0 |
| **18** | 0/0 | 0/0 | 0/0 | 0/0 | 0/1 | 0/1 | 0/0 | 0/0 | 0/0 |
| **19** | 4/3 | 3/3 | 0/0 | 0/0 | 2/2 | 0/1 | 0/2 | 0/0 | 0/0 |

**Table e-3**: **Semiquantitative scores for lower leg muscles of healthy controls.** The fat replacement was scored with the five-point scale by Goutallier *et al.* and STIR intensities were rated on a scale from 0, normal appearance, to 4, severe involvement of entire muscle. TA… tibialis anterior, EHL/DL… extensor halluces/digitorum, Per… peroneus, TP… tibialis posterior, GM… gastrocnemius medialis, GL… gastrocnemius lateralis, SOL… soleus, FHL… flexor halluces longus, FDL… flexor digitorum longus

| **Healthy control** | **TA**  Fat/STIR | **EHL/DL**  Fat/STIR | **Per**  Fat/STIR | **TP**  Fat/STIR | **GM**  Fat/STIR | **GL**  Fat/STIR | **SOL**  Fat/STIR | **FHL**  Fat/STIR | **FDL**  Fat/STIR |
| --- | --- | --- | --- | --- | --- | --- | --- | --- | --- |
| **1** | 0/0 | 0/0 | 0/0 | 0/0 | 0/1 | 0/1 | 0/0 | 0/0 | 0/0 |
| **2** | 0/0 | 0/0 | 0/0 | 0/0 | 0/1 | 0/1 | 0/0 | 0/0 | 0/0 |
| **3** | 0/0 | 0/0 | 0/0 | 0/0 | 0/1 | 0/0 | 0/0 | 0/0 | 0/0 |
| **4** | 0/0 | 0/0 | 0/0 | 0/0 | 0/0 | 0/1 | 0/0 | 0/0 | 0/0 |
| **5** | 0/0 | 0/0 | 0/1 | 0/0 | 0/1 | 0/1 | 0/0 | 0/0 | 0/0 |
| **6** | 0/0 | 0/0 | 0/0 | 0/0 | 0/1 | 0/1 | 0/0 | 0/0 | 0/0 |
| **7** | 0/0 | 0/0 | 0/1 | 0/0 | 0/1 | 0/1 | 0/0 | 0/0 | 0/0 |
| **8** | 0/0 | 0/0 | 0/0 | 0/0 | 0/1 | 0/1 | 0/0 | 0/0 | 0/0 |
| **9** | 0/0 | 0/0 | 0/0 | 0/0 | 0/1 | 0/1 | 0/0 | 0/0 | 0/0 |
| **10** | 0/0 | 0/0 | 0/0 | 0/0 | 0/1 | 0/1 | 0/0 | 0/0 | 0/0 |
| **11** | 0/0 | 0/0 | 0/0 | 0/0 | 0/1 | 0/1 | 0/0 | 0/0 | 0/0 |
| **12** | 0/0 | 0/0 | 0/0 | 0/0 | 0/1 | 0/1 | 0/0 | 0/0 | 0/0 |

**Table e-4:** **The median and quartiles (1^st^ and 3^rd^ Q) of water T1, water T2, fat fraction, total sodium concentration (TSC), inversion-recovery (IR), triple quantum filter (TQF), IR/TSC ratio, and TQF/TSC ratio in different regions.** The subject groups (FSHD versus control) were compared using Wilcoxon rank-sum test (WRST). GM… gastrocnemius medialis, SOL… soleus, TA… tibialis anterior, TP… tibialis posterior

| **MRI measure** | **muscle** | **Controls** | | | **FSHD** | | | **WRST** |
| --- | --- | --- | --- | --- | --- | --- | --- | --- |
|  |  | median | 1st Q | 3rd Q | median | 1st Q | 3rd Q | **p-value** |
| **Water T_1_ (ms)** | **GM** | 1237.4 | 1207.0 | 1272.7 | 1276.9 | 1232.9 | 1398.4 | **0.043** |
|  | **SOL** | 1193.3 | 1184.1 | 1221.9 | 1224.8 | 1185.0 | 1262.2 | 0.186 |
|  | **TA** | 1191.9 | 1175.6 | 1204.2 | 1374.3 | 1197.9 | 1522.1 | **0.020** |
|  | **TP** | 1204.2 | 1188.3 | 1229.7 | 1212.4 | 1173.0 | 1254.3 | 0.777 |
| **Water T_2_ (ms)** | **GM** | 38.5 | 36.8 | 39.2 | 37.5 | 35.7 | 39.6 | 0.570 |
|  | **SOL** | 36.5 | 35.5 | 38.1 | 38.1 | 36.7 | 40.6 | 0.144 |
|  | **TA** | 37.2 | 36.9 | 37.9 | 37.4 | 35.1 | 41.8 | 1 |
|  | **TP** | 35.9 | 35.3 | 36.6 | 35.5 | 34.9 | 37.0 | 0.529 |
| **Fat fraction** | **GM** | 0.040 | 0.032 | 0.046 | 0.302 | 0.058 | 0.584 | **0.0001** |
|  | **SOL** | 0.041 | 0.036 | 0.051 | 0.137 | 0.103 | 0.449 | **0.0094** |
|  | **TA** | 0.033 | 0.026 | 0.037 | 0.300 | 0.222 | 0.499 | **0.013** |
|  | **TP** | 0.034 | 0.029 | 0.043 | 0.158 | 0.053 | 0.184 | 0.113 |
| **TSC (mM)** | **GM** | 20.3 | 18.3 | 21.9 | 24.4 | 15.9 | 41.2 | 0.411 |
|  | **SOL** | 20.7 | 18.7 | 23.7 | 20.8 | 17.4 | 30.8 | 0.738 |
|  | **TA** | 16.8 | 14.8 | 17.4 | 23.2 | 15.4 | 43.7 | 0.165 |
|  | **TP** | 17.6 | 17.1 | 20.4 | 15.7 | 13.7 | 19.7 | 0.105 |
| **IR (a.u.)** | **GM** | 0.461 | 0.401 | 0.478 | 0.465 | 0.347 | 0.798 | 0.681 |
|  | **SOL** | 0.490 | 0.466 | 0.500 | 0.479 | 0.344 | 0.746 | 0.857 |
|  | **TA** | 0.399 | 0.376 | 0.416 | 0.491 | 0.370 | 0.671 | 0.471 |
|  | **TP** | 0.443 | 0.425 | 0.491 | 0.403 | 0.364 | 0.467 | **0.047** |
| **TQF (a.u.)** | **GM** | 0.696 | 0.671 | 0.735 | 0.980 | 0.694 | 1.329 | 0.073 |
|  | **SOL** | 0.631 | 0.587 | 0.678 | 0.831 | 0.581 | 1.883 | 0.247 |
|  | **TA** | 0.726 | 0.693 | 0.796 | 1.378 | 0.965 | 1.950 | **0.019** |
|  | **TP** | 0.685 | 0.577 | 0.692 | 0.652 | 0.516 | 0.697 | 0.433 |
| **IR/TSC** | **GM** | 0.023 | 0.019 | 0.025 | 0.020 | 0.018 | 0.022 | 0.589 |
|  | **SOL** | 0.024 | 0.021 | 0.025 | 0.022 | 0.020 | 0.024 | 0.488 |
|  | **TA** | 0.025 | 0.023 | 0.026 | 0.022 | 0.015 | 0.025 | **0.042** |
|  | **TP** | 0.025 | 0.023 | 0.027 | 0.026 | 0.024 | 0.027 | 0.857 |
| **TQF/TSC** | **GM** | 0.034 | 0.032 | 0.039 | 0.039 | 0.028 | 0.048 | 0.526 |
|  | **SOL** | 0.030 | 0.028 | 0.033 | 0.035 | 0.031 | 0.045 | 0.080 |
|  | **TA** | 0.047 | 0.046 | 0.050 | 0.048 | 0.042 | 0.064 | 0.852 |
|  | **TP** | 0.034 | 0.034 | 0.038 | 0.042 | 0.031 | 0.048 | 0.433 |

**Table e-5:** **Correlations of water T1, water T2, total sodium concentration (TSC), inversion-recovery (IR), and triple quantum filter (TQF).** Relationships of MRI parameters were determined using Spearman correlation coefficients. False discovery rate (FDR) correction was applied for multiple comparisons (p_FDR_). GM… gastrocnemius medialis, SOL… soleus, TA… tibialis anterior, TP… tibialis posterior

| **correlations** | | **r** | **p** | **p_FDR_** |
| --- | --- | --- | --- | --- |
| **Water T_2_ (ms)** | **Water T_1_ (ms)** | 0.4318 | <0.0001 | <0.0001 |
|  | **TSC (mM)** | 0.5177 | <0.0001 | <0.0001 |
|  | **IR (a.u.)** | 0.2263 | 0.0191 | 0.0191 |
|  | **TQF (a.u.)** | 0.3132 | 0.0039 | 0.0043 |
| **Water T_1_ (ms)** | **TSC (mM)** | 0.547 | <0.0001 | <0.0001 |
|  | **IR (a.u.)** | 0.3267 | 0.0010 | 0.0013 |
|  | **TQF (a.u.)** | 0.4238 | 0.0001 | 0.0001 |
| **TSC (mM)** | **IR (a.u.)** | 0.8214 | <0.0001 | <0.0001 |
|  | **TQF (a.u.)** | 0.5386 | <0.0001 | <0.0001 |
| **IR (a.u.)** | **TQF (a.u.)** | 0.4979 | <0.0001 | <0.0001 |


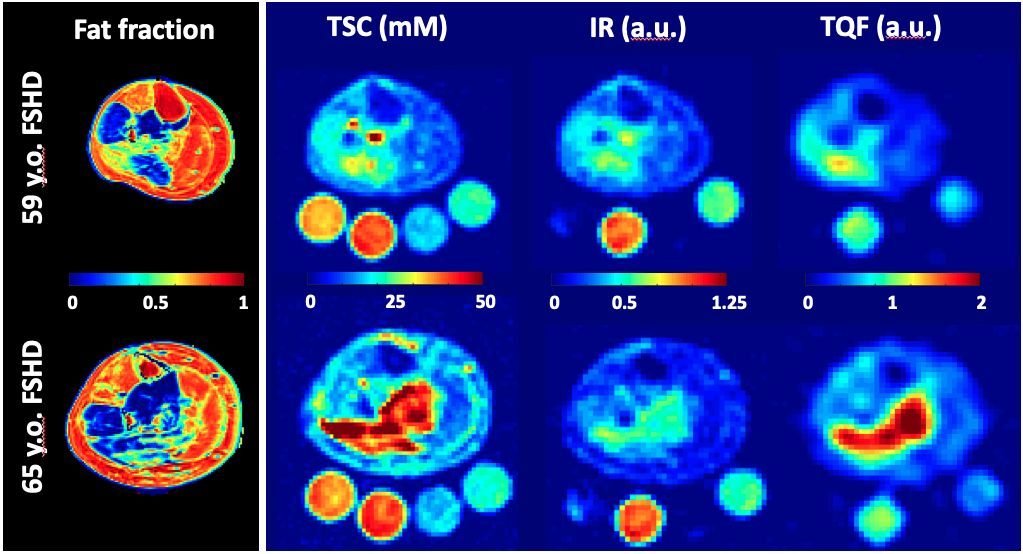


**Figure e-1**: **Examples of fat fraction maps and corresponding sodium maps.** FF derived from the Dixon method as well as tissue sodium concentration (TSC), inversion-recovery (IR), and triple-quantum filter (TQF) signal maps are presented from 59-year-old (#6) and 65-year-old (#5) patients with Facioscapulohumeral muscular dystrophy (FSHD). In completely fat-replaced muscles and the subcutaneous fat, the sodium signals are lower compared to healthy and diseased muscle tissue. The signal in the fat-replaced muscles resembled the signal of the subcutaneous fat. Quantification of the sodium signals in the decomposed gastrocnemius medialis of the 55-year-old patient yielded 11.2 mM TSC, 0.26 a.u. IR signal, and 0.24 a.u. TQF signal, while the subcutaneous fat yielded 10.6 mM TSC, 0.21 a.u. IR signal, and 0.15 a.u. TQF signal.


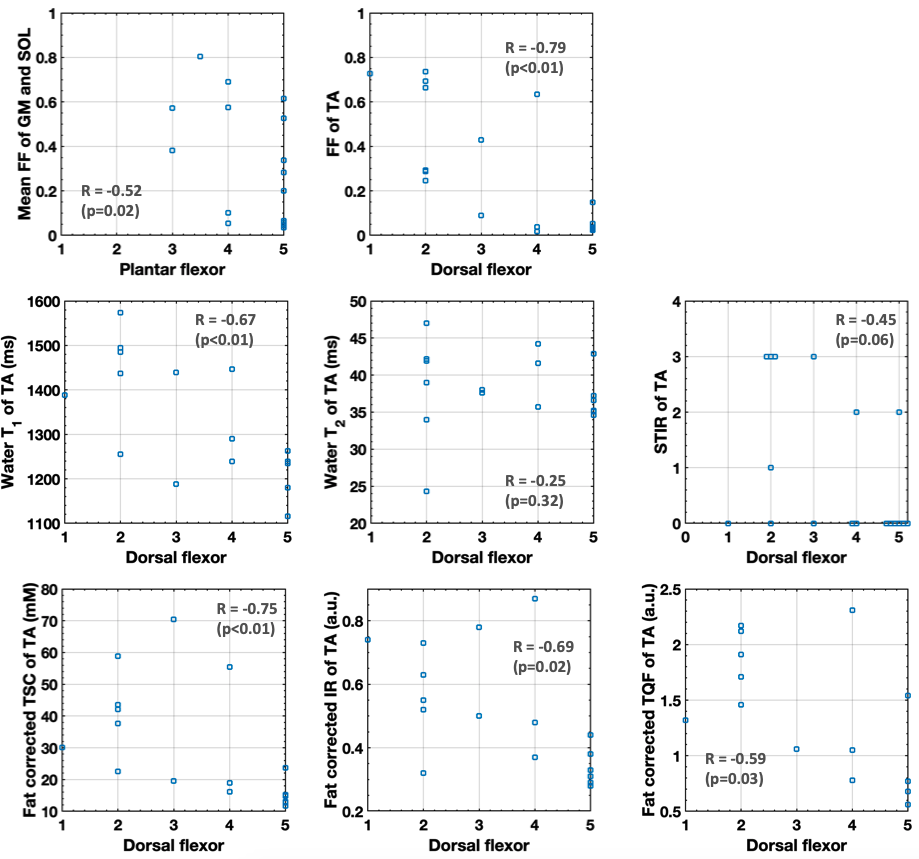


**Figure e-2**: **Relationship of fat fraction and disease activity indices with muscle strength.** For the correlation with plantar flexor strength, a mean FF was calculated by averaging the FF of GM and SOL. Both assessed muscle strengths correlated with the degree of fat replacement. While the TA is the sole muscle involved in the dorsal flexion, the plantar flexor comprises the GM, GL, and SOL. This involvement of three muscles could allow a compensation of an affected muscle by the others and thus explains the weaker relation with the measured plantar flexor strength. All disease activity indices of TA except water T_2_ and STIR were correlated with dorsal flexion. GM… gastrocnemius medialis, SOL… soleus, TA… tibialis anterior


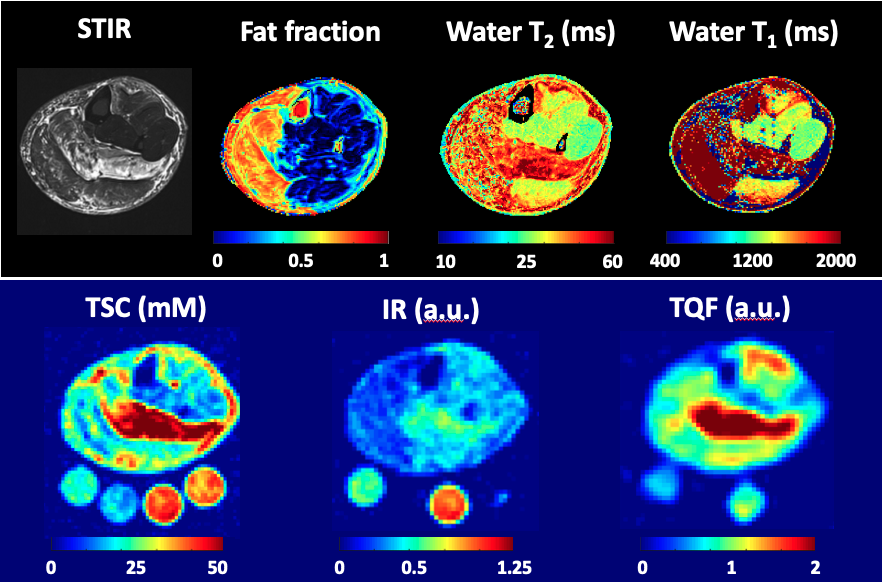


**Figure e-3**: **Case of fat replaced muscles but no paresis in dorsal and plantar flexion of analyzed leg.** This 61-year-old FSHD patient (#15) scored five out of five at the strength test before MR imaging was performed. The GM and SOL were completely or partly replaced by fat, while the rest of the SOL was affected by edema/ inflammation/ necrosis with alterations in all quantitative disease activity indices.
